# Building management and innovation capabilities for global health: a senior executive program

**DOI:** 10.3389/fpubh.2023.1053745

**Published:** 2023-08-29

**Authors:** Andrea M. Prado, Jose Ignacio Sánchez-Gómez, Núria Casamitjana, Marina Espriu, Pratap Kumar, Ben Ngoye, Till Bärnighausen, Magda Rosenmoller

**Affiliations:** ^1^Management and Organizations Department, INCAE Business School, Alajuela, Costa Rica; ^2^Instituto de Investigación de la Universidad de Barcelona, Instituto Salud Global Barcelona (ISGlobal), Barcelona, Spain; ^3^Department of Medicine, School of Medicine and Health Sciences, University of Barcelona, Barcelona, Spain; ^4^Strathmore Business School, Strathmore University, Nairobi, Kenya; ^5^Heidelberg Institute of Global Health, Heidelberg University Hospital, Heidelberg, Baden-Württemberg, Germany; ^6^IESE Business School, University of Navarra, Barcelona, Spain

**Keywords:** global health, management, innovation, training, entrepreneurship, executive, Global South

## Abstract

Accurately approaching the major challenges associated with global health management has become a mandatory key point in the training of medical leaders around the world. The Senior Executive Program in Global Health Innovation Management (SEPGHIM) seeks to provide an answer to the need for innovation and managerial capacity building in Global Health and to address the current detachment between Public Health Organizations and Business Schools. In 2019, SEPGHIM's first edition was led by five prestigious academic institutions on three continents. The first cohort included a total of 27 high-level health professionals and executives from 16 countries with 7–10 years of working experience who participated during the 11 months of the course. The program sought to fill an often-found knowledge gap among health professionals in terms of health innovation, leadership, and management. SEPGHIM relied on multiple pedagogical methods conveyed through a robust theoretical and applied syllabus that included case studies, simulations, guest speakers, debates, site visits, and an executive challenge. The program achieved various results. First, it recruited high-level health professionals that ensured diversity of backgrounds, allowing an exchange of experiences and different ways of addressing global health challenges. Second, it created a network of health professionals for possible future collaborations that can anticipate new trends and opportunities in global health and work together with stakeholders from other sectors. This networking was one of the most highly rated benefits by the students. Finally, the participants expressed great eagerness to recommend the program (4.9 out of 5) to other decision-makers and leaders in the global health field. These results provide positive insights regarding the value of such a training program for senior health professionals.

## 1. Introduction

In a context where economic pressure is increasing healthcare costs (1, 2) while financial resources remain limited, ensuring high-quality healthcare seems challenging (3). Moreover, the still ongoing COVID-19 pandemic and the constant risk of new outbreaks of diseases—as well as the risks that large migrations bring—evidenced the importance of innovation, creativity, and new solutions in global health, hence the need for skilled health professionals to drive innovations (4, 5).

Trained leaders and executives can respond to the increasing global health needs and create the right environment for high-quality care (6). Due to the complex nature of leadership development, the challenge lies in integrating these skills effectively into the national health services' staff training and empowering leadership at a local level (7).

Multiple scholars have acknowledged that management training is needed for improving health systems (3, 7, 8). Nevertheless, business schools are underrepresented in many interprofessional health training plans, creating disparities in the medical curricula, especially for professionals in developing countries, where institutional voids and insufficiency of resources are the norm (3).

When framing global health difficulties in the context of the rich North and poor South proposed by the Brandt Line (an imaginary division), the scope of the problem becomes even wider, as many of the poor nations are forced to face these challenges in a quotidian manner (9). By bringing together professionals from both geographies within the same cohort, there is not only an opportunity to enrich and provide them with a full-picture vision but also to generate shared value and networking (10).

To better understand the needs of senior-level professionals and executives working on Global Health, the Barcelona Institute for Global Health (ISGlobal), a Senior Executive Program in Global Health Innovation Management (SEPGHIM) partner, performed an online survey in 2018, gathering insights from 126 health professionals from 44 countries interested in expanding their knowledge and skills in leading innovation.

Most respondents (63.5%) were eager to participate in education training combining Global Health, Innovation, and Management. Preferred formats were mostly short courses (45%) or executive programs (25%), with very low interest for MBAs, master's, and Executive MBAs (<8%). Blended programs, including both online and face-to-face sessions, were largely selected (70%) against full-online programs (20%). Finally, 82% of respondents preferred a part-time format against full-time (11%), and 84% expressed a strong interest in working on an executive challenge.

The above needs assessment survey results do not match the current market educational offer, since out of the 52 active educational programs identified worldwide on Global Health Innovation and/or Management, only four provide blended content, and the rest include long-term programs (e.g., master's and MBAs), limited content, unique format, and location.

These data were complemented with a set of semi-structured interviews with innovators and executives responsible for innovation or human resource departments from leading industries. Interviewees were partners within the European Institute of Innovation and Technology (EIT) Health Network, an alliance of leading academic institutions and industries in health innovation financed by the European Union. Insights were refined in a Design Thinking workshop held at IESE Business School with a wide range of actors and stakeholders in 2018. The results were updated by IESE in a second survey in 2020.

Among the gaps exposed by the survey and the interviews was how, in the fast-changing Global Health context, the need for managerial and innovation capacity building is more critical than ever, highlighting the key role of a better approach to executive education. However, the traditional educational offer is directed to limited targets (e.g., master's), has geographical limits (low number of programs including cross-continent partnerships between Latin America, Africa, and Asia), a unique condition focus (e.g., malaria), and its content usually does not cover all aspects of global health, innovation, and entrepreneurship.

With these insights, SEPGHIM aims to bridge the persistent gap between higher education and innovation (SIA2021-27^1^) by bringing together five world-class academic institutions—supported by EIT Health—to launch SEPGHIM, a fellowship program with a curriculum tailored to provide solutions to the challenges identified. The program aimed to have global outreach through a broad network that would connect future fellows and current global health leaders with institutions. The support of EIT Health was vital to the development of the program, which realized the importance of connecting with Global Health partners in the Global South to achieve its goal of fostering business, research, and education to bring innovative and real-world health solutions to markets.

## 2. Context

From the perspective of business, SEPGHIM was led by IESE Business School in Barcelona, INCAE Business School in Costa Rica, and Strathmore Business School in Kenya. Jointly, these institutions supported the initiative by building a solid program to address management, leadership, and innovation. These institutions also have a trajectory related to managing healthcare programs. IESE runs the Center for Research in Healthcare Innovation Management (CRHIM), INCAE leads the Central American Healthcare Initiative (CAHI), and Strathmore Business School offers the Managing Healthcare Businesses (MHB) program (11).

On the Global Health side, SEPGHIM was led by ISGlobal, the Barcelona Institute for Global Health associated with the University of Barcelona and the University Pompeu Fabra in Spain, and the Heidelberg Institute of Global Health (HIGH) associated with the University of Heidelberg in Germany. Both institutions are leading partners of TropEd, the network for education in international health.^2^ These two institutions collaborated with the business schools mentioned above to design and deliver SEPGHIM.

Regarding the target audience of the program, it is intended to be broader than just clinician leaders. SEPGHIM targets **senior executives in the field of Global Health with 7–15 years of working experience**, such as decision-makers, executives, and leaders in global health-related fields, such as health providers and authorities, initiatives by international health organizations, the industry (pharma, med/biotech, and IT), the healthcare system, ministries of health, and leading non-governmental organization activities.

Since the program aims to develop a multidisciplinary approach to solving health problems in various geographical settings, a special effort has been made to **recruit participants with various cultural, geographical, gender, institutional, and professional backgrounds**.

The initial recruitment for SEPGHIM's first generation was through a social media campaign among the different official sites and alumni communities of the academic institutions involved. Registration was formalized on SEPGHIM's official website, and subsequently, an Evaluation Committee, composed of at least one representative of each partner, remotely assessed and scored the applicant's adequacy to the program according to pre-established criteria regarding the level of education, working experience, and level of expertise, measured on years of experience in global health, innovation, and/or management. We also looked at their likely impact, including their motivation and the type of project they would be working on throughout the program, as well as their background and geographical location (see Table 1).

**Table 1:** SEPGHIM fellows' demographics and affiliations.

	**Fellowship 2019**	
	**Fellows**	**%**
Total	27	100
**Region**		
Europe	9	33
Central America	6	22
Africa	5	19
North America	3	11
Asia	2	7
South America	2	7
**Gender**		
Male	10	37
Female	17	63
**Affiliations**		
Academia/Research institution	7	26
Non-governmental organization	7	26
Healthcare providers	5	19
Pharma/Medtech	3	11
Start-up/Biotechnology	3	11
Policymakers	2	7

Once completed, SEPGHIM 2019 retained high-level health professionals and executives from 16 countries with a working experience of 7–10 years. The cohort had a total of 27 participants out of 53 applicants from diverse settings: public, private, and non-governmental organizations (NGOs).

The limited number of participants could be considered a limitation of the SEPGHIM program; rather, this constitutes an opportunity to guarantee a high level of selection and therefore ensure rich exchanges among participants, especially for the executive challenge, including peer feedback sessions in small groups and individual mentoring with high-level experts.

The group's composition provided perspectives from nations such as Brazil, the Netherlands, Sri Lanka, and Uganda. It also managed to have significantly more female than male participation. This statistic addresses the importance of women leaders, as stated again by the WHO in 2019: healthcare is delivered by women but led by men (12). Thus, SEPGHIM trains a multidisciplinary group of high-profile professionals and decision-makers—many of them women—equipping them to address global health challenges.

## 3. Sepghim program

### 3.1. Modules and learning objectives

The SEPGHIM program is based on a learning journey where the blended methodology (face-to-face sessions and online sessions) aims to (1) foster **innovation and entrepreneurial capacity** for global health executives, (2) increase the ability of learners to use new **digital technologies** to support the development of new solutions to global health challenges, and (3) strengthen a **sustainable global health network** across three continents.

Each module hosts a half-day open co-creation Workshop, bringing together citizens, local experts, and SEPGHIM participants to create concrete solutions to address global health challenges. In addition, the different modules revolve around hands-on experiences (onsite visits to the local innovation ecosystem), working group exercises, simulation, interaction with the main stakeholders of the sector (panels and inspirational talks), and networking events, including local experts and SEPGHIM alumni.

These modules are complemented with online pre- and post-module activities and an executive challenge running throughout the program to address key competencies such as digital and data literacy, health system awareness, management and leadership, entrepreneurship and multidisciplinary skills, innovation, critical thinking and decision-making, citizen-oriented skills, and communications abilities.

The **first** module delivered by the Heidelberg Institute of Global Health (HIGH) in Germany focused on *Understanding the Health Challenges in the Global Context*. The module's objectives were to strengthen the ability of each participant to lead change in global health across policies, sectors, and disciplines while addressing current and potential future global health challenges. Participants acquired tools to help them determine the innovation potential of global health ecosystems and create feasible strategies in the broader context of political and economic factors.

This initial module addressed topics of global health in the context of the Sustainable Development Goals: innovation, integration of health prevention and promotion, digital health strategy for health system strengthening, and opportunities for intersectoral collaboration. In line with the program, site visits were organized to provide participants with the opportunity to experience the local innovation ecosystem. Students visited BASF, a chemical company that combines business and social perspectives to create shared value. The focus of the BASF executive presentation was on food fortification and strategies to tackle nutrition deficiencies.

The second module took place in Costa Rica and was hosted by INCAE Business School. This module, called *Leading Innovation and Change*, explored how entrepreneurship—and other business models—can contribute to global health and the challenges it faces in the context of middle- and low-income countries. Students discussed how to use ethnographic methods and develop business models to serve communities at the base of the pyramid.

The specific objectives of this module are to (1) explore how an entrepreneurial mindset can drive global health innovation, (2) acquire management tools and skills to integrate stakeholders' needs and resources to support the success of their projects, and (3) analyze innovative business models to serve the base of the pyramid.

Some of the topics in this module were discussed with a case study methodology, potentiating the students' critical thinking. Other sessions included conferences on challenges for entrepreneurship in the Latin American context and on the Costa Rican healthcare system, which is internationally recognized for its universal coverage and the strength of its primary level of attention. Intrapreneurship was covered through a simulation where students had to lead a change initiative within an organization.

Students also visited one of Costa Rica's primary care clinics (EBAIS) and attended a panel discussion with prestigious non-governmental organizations working with vulnerable populations and their health challenges. Finally, a local scientist and entrepreneur shared the opportunities and challenges of doing first-class clinical research—finding the cure for pancreatic cancer—in a middle-income country like Costa Rica.

The third module was held in Nairobi, Kenya, led by Strathmore Business School, and was oriented on *Developing New Services and Products in Global Health*. This module developed effective strategies to fully leverage the potential of digital transformation in Global Health, expanding understanding of operational challenges facing global health organizations to achieve breakthrough services, and leveraging opportunities in innovative financing mechanisms.

The specific learning objectives for this module are to (1) develop effective strategies to fully leverage the digital transformation in global health, (2) expand the understanding of the operational challenges facing global health organizations to achieve breakthrough service, and (3) leverage opportunities in Innovative Financing Mechanisms.

Under this framework, fellows learned about the digital startup and innovation ecosystem in Kenya, universal access to healthcare, virtual training for health professionals, and the role of impact investment in global health innovation. The former encouraged the participants to place themselves in the shoes of entrepreneurs and medical leaders to fully understand how diverse challenges developed in different contexts on the African continent. Examples of live cases were *Access Afya*, a primary healthcare social enterprise building affordable, convenient, and effective access to healthcare for Kenyan communities, and *m-Tiba*, a mobile phone platform that connects people, payers, and providers in the healthcare sector 24/7, among many others.

A visit to SHOFCO, an organization that catalyzes large-scale transformation in urban slums with critical services for people in Kibera and Mathare, provided participants with a real-life experience of challenges in a complex context. “*Professionally interesting and personally touching. A great opportunity to know the reality of living conditions for many people in urban areas of low-middle income*.”

The fourth module engaged “*Getting things done: the art of implementation*,” led by the IESE Business School with the collaboration of ISGlobal in Barcelona, focusing on leadership and managing change and implementation, and communication tools. The module was developed under three specific learning objectives based on (1) reflecting on your leadership style and acquiring tools to enhance communication with your management team; (2) developing new negotiation skills and deploying them in collaborative and competitive situations; and (3) strengthening project management skills for strategic implementation.

Through highly interactive cases around global economic contexts, participants took active roles in presentations and small group work. Field visits included the Innovation Unit of Hospital Sant Joan de Déu, one of the most innovative pediatric hospitals in Europe. A final panel brought together experts from the Agency for Healthcare Quality and Assessment of Catalonia (AQuAS), ISGlobal, the Spanish Parliament, and Medicos Sin Fronteras (MSF Spain), discussing challenges and opportunities at different levels of governance, reviewing relevant stakeholders in the global health context, their roles, and how to strengthen their alignment and cooperation.

All modules were delivered with 1 month of self-learning in between to allow participants to internalize the new knowledge and skills and apply them to their own personal projects.

### 3.2. The executive challenge

**Figure 1:**
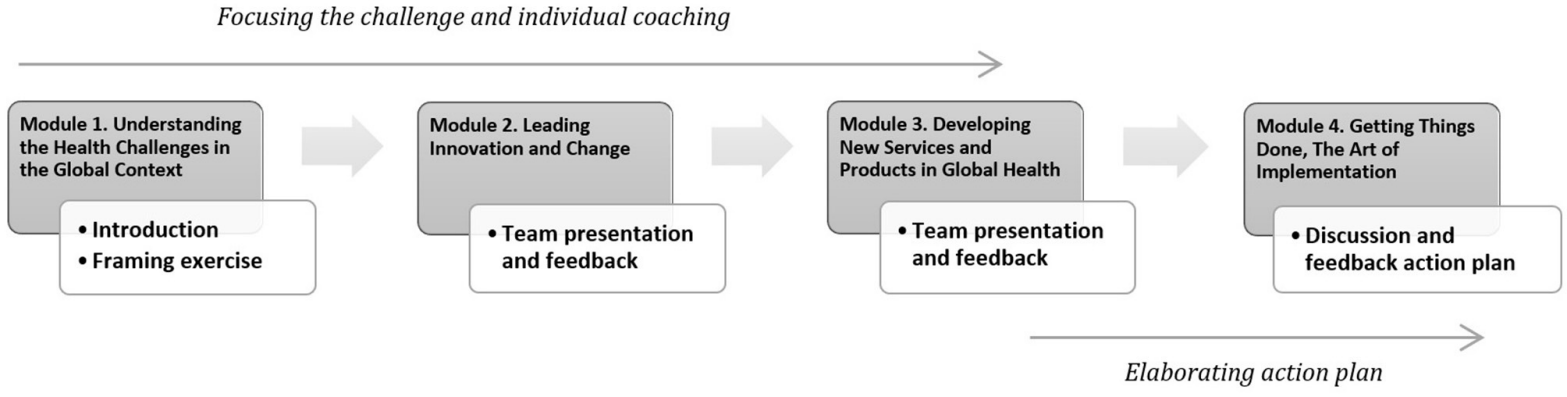
Progression steps of the executive challenge during and in-between modules.

The executive challenge aims to provide participants with the opportunity to address relevant real-time strategic, operational, or personal business challenges and discuss them in teams of eight participants with complementary expertise and backgrounds, enabling interesting and productive discussion. The executive challenge included sessions of mentoring and individual coaching with the SEPGHIM faculty and additional relevant experts (see Figure 1).

It included a variety of initiatives in different sectors for instance, an African executive sought to strengthen the Nigerian primary healthcare services by improving data management systems and enhancing logistics and supply chain management systems. Other challenges had to do with private enterprises. For example, a proposal presented by a Lithuanian entrepreneur sought to launch a new medical device to monitor vitamin D levels and stimulate its synthesis while protecting from harmful radiation and erythema. Another challenge was developed by an NGO leader from Spain, aiming to address the upgrading and expansion of an artificial intelligence platform to improve the diagnosis of infectious diseases in low-income areas with difficult access to health facilities.

## 4. Results

The assessment of SEPGHIM was performed at different stages of the implementation of the program using quantitative (satisfaction module-specific online and program surveys) and qualitative methodology (interviews with learners and faculty). To measure the impact of the program (on the economic, educational, and societal levels), evaluate the SEPGHIM program, and monitor the learners' involvement, a multi-level evaluation was conducted based on the Kirkpatrick model.

Regarding levels 1 and 2 of reaction and learning of the Kirkpatrick model, learners answer an online survey before and after the program to assess the change in their knowledge, skills, and attitudes in global health, innovation, and entrepreneurship (GHIE). The software Evolute Tricuspoid 2.0 was used to analyze entrepreneurship skills and attitudes, measuring the evolution of learners' creative tension, meaning the difference between their current perceived level of competence and the level they would like to achieve. Learners should show less creative tension after the course than at the beginning.

A post-course satisfaction survey assesses various aspects: overall satisfaction (>80% highly satisfied), achievement of learning objectives and content (80%), creative tension of entrepreneur competencies and skills (30% decrease), likelihood of recommending the course (80%), intention to pursue another innovative project or continue with the challenge (60%), satisfaction with the executive challenge plan (80%), knowledge increase (35%), skills and attitude enhancement (20%), and learners benefiting from mentoring (80%).

Qualitative feedback through interviews with students and faculty during the ongoing modules was a tool to enable potential adjustment between modules. Meanwhile, levels 3 and 4 of the Kirkpatrick model regarding behavior and results were measured through online follow-up surveys to assess the impact and individual progress. The overall rating for this first edition of the program was 4.5 out of 5.

The exit survey also asked students about their perceived benefits. Like in other innovation and entrepreneurship programs (10), accessing a network of health professionals was particularly valued by participants. The second most important benefit perceived was learning about new trends and opportunities in global health, as this knowledge supports their innovation skills and allows them to be at the industry's forefront. Finally, participants valued the opportunity to work together with different stakeholders, confirming the importance of a diverse cohort (see Table 2).

**Table 2:** Perceived benefits by SEPGHIM participants.

	**Score over five points**
**Criteria**	
Connect to a network of health leaders and innovators	4.5
Anticipate new trends and opportunities in global health	4.3
Work together with stakeholders from other sectors	4.2
Advance your professional career	4.1
Implement and manage an innovation project in global health	4.1
Motivate and manage a team	4.1
Consider alternative business models to deliver global health solutions	4.1
Address global health challenges with broader approaches	4.0
**Testimonials of the participants**	
“The best part of Module 1 is the lecture on personnel management and the introduction to the Executive Challenge”	Participant of module 1
“I discover new topics such as ethnographic research very valuable. Also, the boot camp visiting health providers in Costa Rica, and the entrepreneurship testimonies. I also love the class of literature and leadership”	Participant of module 2
“The best was the practical examples given by the entrepreneurs. I have learned a lot discussing the challenges and trying to connect that with the challenges that I'm facing right now founding my own company”	Participant of module 3
“The best of the module were the sessions on soft skills, e.g., communication, power dynamics, and agile project management”	Participant of module 4

Overall, participants agreed that SEPGHIM was groundbreaking for their personal growth and added great value to their professional careers, increasing their ability to innovate in a complex and fast-changing environment. They also expressed great willingness to recommend this program (4.9 out of 5) to decision-makers and leaders in global health, denoting their perception that the program would be useful for their peers.

A year after concluding the program, IESE performed follow-up evaluations among SEPGHIM alumni. The results show that 60% increased responsibility and salary, and 80% implemented their executive challenge. Respondents gave an evaluation of 4.6 out of five on the usefulness of the knowledge and skills learned in the program.

## 5. Discussion

Effectively addressing global health challenges will require managing limited resources and innovating health products and services, as well as business models. Academic programs that seek to train professionals working in global health must complement health-related education with management capabilities—including the ability to innovate. As university departments often work in silos, many executive programs focus on a single academic discipline. A program such as SEPGHIM combines the expertise and knowledge of both Health and Business schools, enlarging the set of capabilities that participants can acquire to tackle global health challenges.

In addition, executive programs for professionals working on global health will likely benefit from more geographically diverse cohorts. Even though middle- and low-income countries might share similar health challenges, their institutional environments and solutions could vary significantly. Programs such as the CAHI at INCAE (3) or the MHS at Strathmore (11) are more regional than global in terms of participants' origin. Instead, SEPGHIM recruits from a geographically diverse pool of candidates through its different academic partners located on three continents. A geographically diverse cohort provides the opportunity for exchanging knowledge and broadening perspectives, supporting the development of innovations that can improve health outcomes.

The Global Health field is not led by governments alone. The private sector, civil society, and multilateral organizations are all engaged in efforts toward improving health in low- and middle-income countries. Intersectoral partnerships become essential for these efforts to succeed, as, among other things, they help accelerate the development and deployment of new medical technologies and treatments (13). Collaborative work could represent the best way to increase affordable access to high-quality healthcare and, ideally, universal health coverage (14, 15). As shown by SEPGHIM, executive programs that provide the opportunity for representatives of multiple sectors to connect and network are highly valued by participants.

Countries around the world are seeking to increase their preparedness to respond to health emergencies and pandemics by building stronger health systems that are better equipped to handle these crises (8, 16). In addition, problems related to finances and inequality (North-South), fragmented systems, access to knowledge, and trained human resources need to also be addressed to set the pace for an optimum health system (17). Through programs such as SEPGHIM, academia could contribute to building the capabilities of health professionals and business executives to achieve a more equitable, resilient, and innovative-oriented health system.

## Data availability statement

The raw data supporting the conclusions of this article will be made available by the authors, only under request.

## Ethics statement

Ethical review and approval was not required for this study in accordance with the local legislation and institutional requirements. Written informed consent from the program evaluation focus group participants was not required to participate in this study in accordance with the national legislation and the institutional requirements.

## Author contributions

All authors listed have made a substantial, direct, and intellectual contribution to the work and approved it for publication.

## Footnotes

^1^The SIA 2021-2017 statement regarding the “**innovators and business creators lack the needed entrepreneurial and innovations skills**” is confirmed by the educational offer gap identified through the Market Research conducted in 2018 and updated in July 2020.

^2^tropEd - https://troped.org/.
